# Thiazole–amino acids: influence of thiazole ring on conformational properties of amino acid residues

**DOI:** 10.1007/s00726-021-02974-0

**Published:** 2021-04-10

**Authors:** Monika Staś, Małgorzata A. Broda, Dawid Siodłak

**Affiliations:** 1 Faculty of Chemistry, University of Opole, 45-052 Opole, Poland; 2 Institute of Organic Chemistry and Biochemistry of the Czech Academy of Science, Flemingovo Náměstí 2, 166 10 Praha 6, Czech Republic

**Keywords:** Thiazole, Non-standard amino acids, Conformational analysis, Ramachandran map, Hydrogen bond, DFT

## Abstract

Post-translational modified thiazole–amino acid (Xaa–Tzl) residues have been found in macrocyclic peptides (e.g., thiopeptides and cyanobactins), which mostly inhibit protein synthesis in Gram + bacteria. Conformational study of the series of model compounds containing this structural motif with alanine, dehydroalanine, dehydrobutyrine and dehydrophenylalanine were performed using DFT method in various environments. The solid-state crystal structure conformations of thiazole–amino acid residues retrieved from the Cambridge Structural Database were also analysed. The studied structural units tend to adopt the unique semi-extended β2 conformation; which is stabilised mainly by N–H⋯N_Tzl_ hydrogen bond, and for dehydroamino acids also by π-electron conjugation. The conformational preferences of amino acids with a thiazole ring were compared with oxazole analogues and the role of the sulfur atom in stabilising the conformations of studied peptides was discussed.

**Supplementary Information:**

The online version contains supplementary material available at 10.1007/s00726-021-02974-0.

## Introduction

The biological activity and metal-binding properties of proteins and peptides depend on their conformation (Giri Rao and Gosavi 2016). The capability of changing ligand conformation to improve binding affinity in proteins is one of the biomolecular engineering tools crucial for drug discovery and design (Lassila 2010; Gagné et al. 2012; Boehr et al. 2018; Ding et al. 2020; Aguesseau-Kondrotas et al. 2019). Mapping the conformational space of small peptide fragments is also an important prerequisite in decoding the protein folding process and understanding protein structure (Culka et al. 2019; Culka and Rulíšek 2019, 2020; Ganesan and Paranthaman 2020; Chahkandi et al. 2014). According to the funnel landscape theory, the side chains are primarily responsible for folding diversity. On the contrary, the backbone-based theory postulates that the universality of the osmolyte effect points to the conclusion that self-organisation takes place mainly in the main chain (Rose et al. 2006; Dill et al. 2008). A recent ab initio study confirmed that such a small unit as tripeptides fragments composed of standard amino acids already exhibit a small tendency to adopt a particular secondary structure (helical or extended) (Culka et al. 2019). Regardless of the chosen theory, it is sure that the conformational preferences of amino acid residues or short peptide fragments have a high impact on the overall secondary structure. Studies on unusual (or non-native) amino acid residues can further deepen our insight in the understanding native structure of peptides, predict it and/or design peptides with specific characteristics. Of special interest are azole-based peptides, due to their unique conformation and high propensity to metal complexation such as copper, calcium, or silver (Bertram and Pattenden 2007; Cusack et al. 2002; Gahan and Cusack 2018).

Previous studies have shown that modifications of peptide main chain or side chain as *N*-methylation (Siodłak et al. 2006, 2008, 2012; Broda et al. 2005, 2009), *C*-terminal ester bond (Siodłak et al. 2010, 2011; Siodłak and Janicki 2010), dehydration of side chain (Buczek et al. 2014), cyclization (Staś et al. 2016a), and many more (Jwad et al. 2020; Gil et al. 2009; Paranthaman 2018) have a considerable influence on preferred conformation. This includes the introduction of a five-membered heterocycle, such as oxazole (Siodłak et al. 2014a; Staś et al. 2016a, b) or thiazole, into a peptide main chain as an isosteric replacement of amide group. Peptides that contain heterocyclic amide isosteres are usually more rigid than the corresponding homodetic cyclic peptide (Jwad et al. 2020; Abbenante et al. 1996). Their aromatic character forces the ring atoms into the coplanar arrangement. As a consequence, the flexibility of the peptide main chain decreases, and the overall conformation is more constrained. It often changes the intra- and intermolecular interactions pattern and affects the overall properties of the compound (Schärfer et al. 2013; Reid et al. 2014; Kheirjou et al. 2014).

In nature, heterorings are obtained in post-translational modifications from standard amino acid residues such as threonine, serine, and cysteine (Metelev and Ghilarov 2014). They occur in highly modified macrocyclic peptides; thiopeptides or cyanobactins (Bagley et al. 2005; Jin 2011). In many cases, these compounds have promising anti-tumour, anti-bacterial, or anti-malaria activities (Davyt and Serra 2010). Residues with thiazole ring have the most diverse side chains among other rings (thiazoline, oxazole, and oxazoline). Literature survey reveals that in natural compounds they can be found as thiazole-*glycine* (Kai et al. 2012; Debono et al. 1992; Engelhardt et al. 2010; Jüttner et al. 2001)*, alanine* (Kai et al. 2012; Bagley et al. 2000; Castro Rodríguez et al. 2002; Engelhardt et al. 2010)*, valine* (Bagley et al. 2005; Hughes and Moody 2007; Bertram and Pattenden 2007; Jüttner et al. 2001)*, serine* (Zhang et al. 2009)*, methionine* (Jüttner et al. 2001)*, threonine* (Davyt and Serra 2010)*, leucine* (Davyt and Serra 2010)*, isoleucine* (Jüttner et al. 2001)*, asparagine* (Zhang and Liu 2013; Young and Walsh 2011)*, phenylalanine* (Davyt and Serra 2010)*, dehydroalanine* (Aoki et al. 1991)*, dehydrobutyrine* (Kai et al. 2012; Walsh et al. 2010) and other (Bagley et al. 2005; Jin 2011). The important feature of the thiazole ring is the presence of the *sp*^*2*^ sulfur atom which has a larger size than carbon, nitrogen, or oxygen, and its ability to engage in donating *n*_o_ → σ* interaction (σ-holes) with the lone pairs of neighbouring heteroatoms. It can lead to conformational and steric effects unique for S-containing heterocycles (Dudkin 2012). Sulfur atom in a heteroaromatic ring may also act as a Lewis acid or electrophile and despite its electronegativity, interacts with electron donors, particularly with oxygen or nitrogen atoms, and π-systems. Sulfur-containing heterocycles may also participate in attractive non-bonding interactions that control the molecular conformation. This may improve binding affinity, independently on ligand-target contacts, and enhance selectivity; at the same time mitigating off-target toxicity and/or metabolic modification. Examples can be found in the study of Beno and co-workers (Beno et al. 2015). The energy of S⋯O interaction between thiophene or thiazole and carbonyl oxygen is comparable to a typical hydrogen bond (Murray et al. 2008; Chahkandi and Chahkandi 2020; Aliakbar Tehrani and Fattahi 2013). Thiazole derivatives are widely used in pharma industry (Ilardi et al. 2014). Sulfur-containing ligands improve the inhibitor activity for thrombosis Factor Xa (FXa) and selectivity as for MMP-13 inhibitors (Metalloproteinase) therapeutically useful for curing osteoarthritis (Zhang et al. 2015). Replacement of the amide group by 5-membered heterocycles (thiazole, oxazole, and imidazole) leads to a stronger interaction between the ligand and Mg^2+^ ions of HIV-1 integrase (Le et al. 2010).

**Fig. 1:**
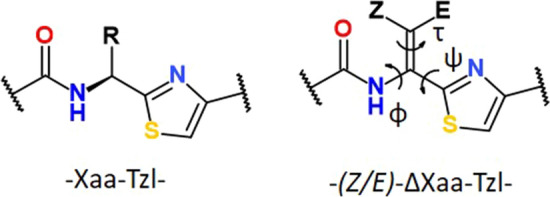
Schematic formula of thiazole–amino acid (-Xaa–Tzl-) and thiazole–α,β–dehydroamino acid residues (-(Z/E)–∆Xaa–Tzl-)

The aim of this work is to provide an in-depth analysis of conformational properties of thiazole-containing amino acids (Xaa–Tzl), where the thiazole ring is in place of the C-terminal amide group (Fig. 1). The simplest and most common naturally occurring residues with thiazole ring were chosen; alanine (Ala–Tzl), dehydroalanine (ΔAla–Tzl), and Z–dehydrobutyrine ((*Z*)–ΔAbu–Tzl). In addition, dehydrophenylalanine (ΔPhe–Tzl) was analysed, the most often studied dehydroamino acid, due to the easily accessible synthesis of both geometric isomers *Z* and *E*. In particular, thiazole–α,β–dehydroamino acid residues are interesting as they combine together two structural motifs; the double bond between α and β carbon atoms and thiazole ring (Siodłak 2015; Jaremko et al. 2013). The results obtained from DFT study were confronted with the data for crystal structures presented in the Cambridge Structural Data Base (Groom et al. 2016). The comparison with conformational properties of oxazole and oxazoline amino acid analogues is also mentioned.

## Computational details

**Fig. 2:**
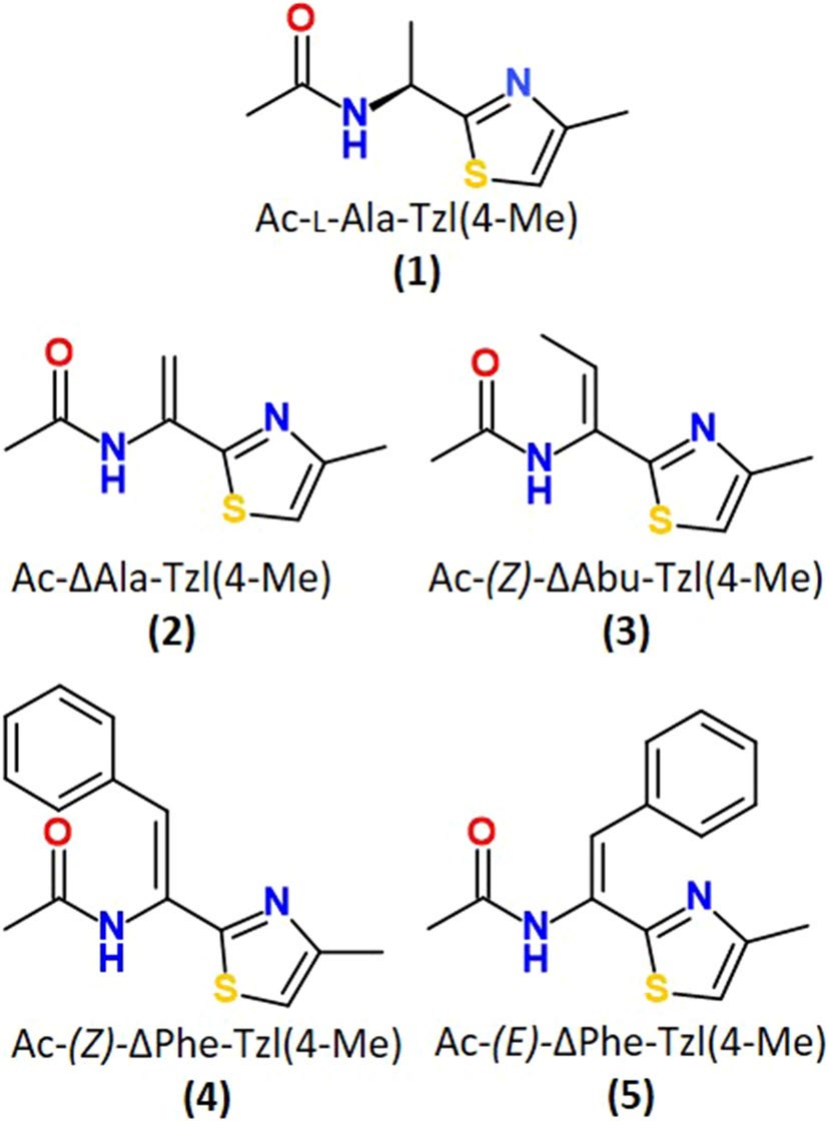
Schematic formula for the studied compounds (1–5)

The conformational properties of the following molecules: Ac–l–Ala–Tzl(*4*-Me) (**1**), Ac–ΔAla–Tzl(*4*–Me) (**2**), Ac–(*Z*)–ΔAbu–Tzl(*4*–Me) (**3**), Ac–(*Z*)–ΔPhe–Tzl(*4*–Me) (**4**), and Ac–(*E*)–ΔPhe–Tzl(*4*–Me) (**5**) were modelled by DFT method (Fig. 2).

Conformational maps for Ac–l–Ala–Tzl(*4*–Me) (**1**) and Ac–ΔAla–Tzl(*4*–Me) (**2**) in gas phase (partial optimization with constrained torsion angles, φ, and ψ, change with the step of 30 degrees), chloroform, and water (single-point calculations) were calculated. Full optimisation was performed for all found local minima. The dehydroamino acids are achiral and each conformation (φ, ψ) has related pair (− φ, − ψ) with the same energy but with opposite torsion angles so that only half of the map is needed to be calculated. Calculations were performed for molecules with the *trans-*amide bond. For the compounds (**3–5**), the minima were calculated on the basis of the map for **2** as well as the minima of corresponding oxazole-amino acids (Siodłak et al. 2014a). The conformers' names are based on the Scarsdale nomenclature (Scarsdale et al. 1983; Hudáky et al. 2004).

Based on our previous studies (Siodłak et al. 2014b) the meta-hybrid M06-2X/6–311++G(d,p) (Zhao and Truhlar 2008) level of theory was chosen. To estimate the solvation effects on the conformations, calculations were also conducted using a self-consistent reaction field (SCRF) with the SMD method (Kang et al. 2011; Kang and Park 2014). The Gaussian 16 package was used (Frisch et al. 2016). The NBO analysis was performed using the same method and basis set as mentioned before (Weinhold and Landis 2001). Frequency analyses were carried out to verify the nature of the minimum state of all stationary points and to calculate the zero-point vibrational energies (ZPVEs). The expected population (*p*) of the conformers at a temperature of 300 K (where RT = 0.595 kcal/mol) was calculated (Hudáky and Perczel 2008; Hruby et al. 1997). Interaction energy in gas phase and water as a solvent between water molecules and oxazole/thiazole was investigated using molecular dynamic and density functional theory. The initial geometry of oxazole and thiazole molecules was created in GaussView6 program (Dennington et al. 2016) and their structures were optimised. Molecular dynamic simulation, 10 ns at 300 K, in explicit water was set up for both rings. Periodic boundary was used. The rings were solvated in a periodic rectangular box filled with water (TIP3PBOX 12) using TLeap. The structures were equilibrated with the Amber14 package (Case et al. 2014) using supplied general amber force field (GAFF) for the rings and “ff14SB” force filed for water. Atomic charges were obtained from the R.E.D server (Bayly et al. 1993; Vanquelef et al. 2011; Dupradeau et al. 2010). After a sequence of restrained minimisations and heating, 100 ps equilibration dynamics was performed at 300 K without any restraints. The 200 initial structures obtained by taking snapshots from the MD trajectory at 5 ps intervals with the makea and xshell programs. The first solvation shell was considered in the distance of 2.5 Å. The DFT optimisation for obtained complexes was performed in gas phase and implicit water (SCRF/SMD) (Kang et al. 2011; Kang and Park 2014) with the M06-2X/6–311++G(d,p) level of theory. All optimised complexes without imaginary frequencies were analysed further.

## Results and discussion

### Thiazole–alanine

**Fig. 3:**
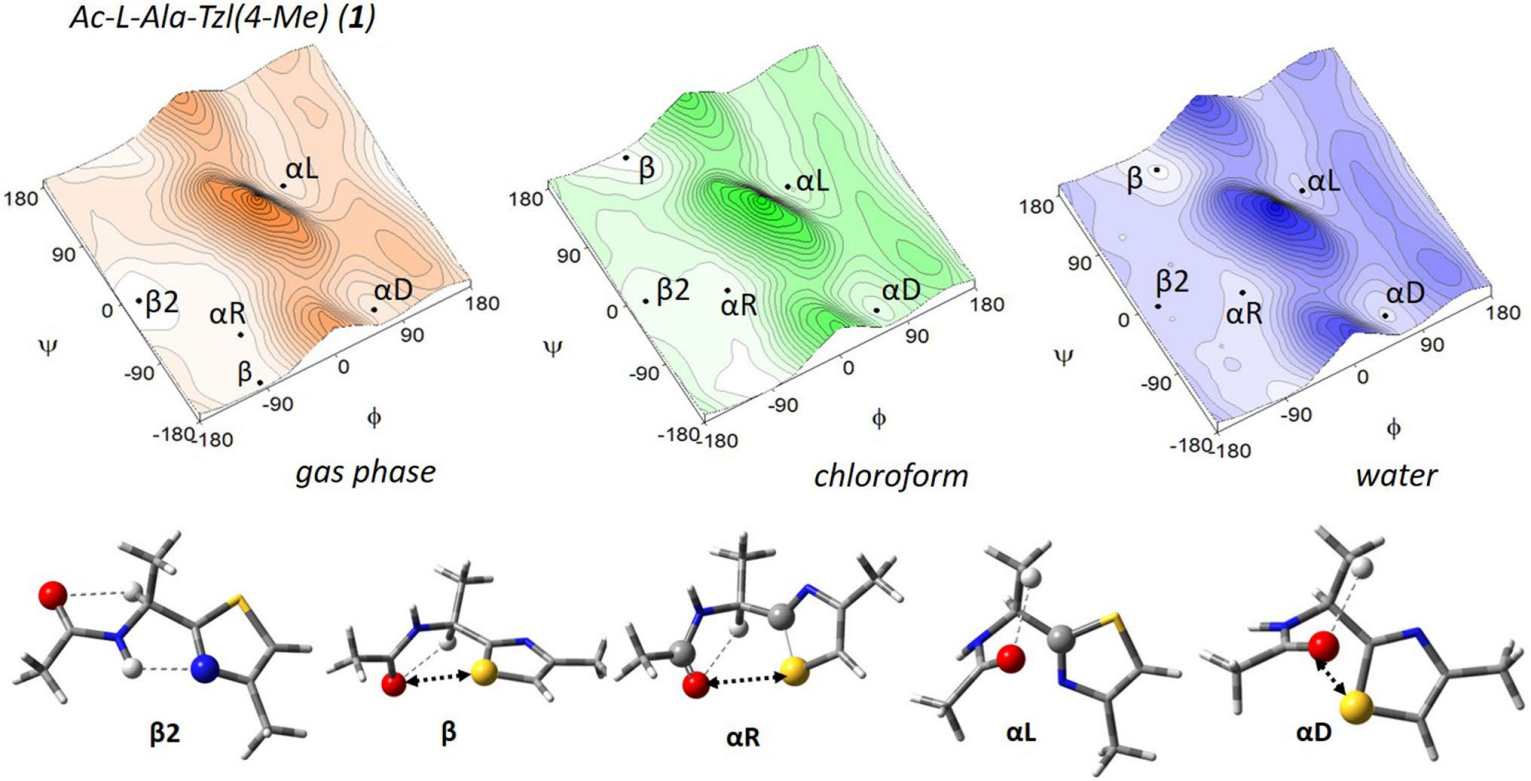
The potential energy surfaces E = f(φ,ψ) for Ac–l–Ala–Tzl(4–Me) (1) calculated by M06–2X/6–311++G(d,p) method in various environment. Energy contours are plotted every 1 kcal/mol. Conformations optimised in chloroform with most important stabilising internal forces; hydrogen bonds (⋯) and electrostatic interactions (◄⋯►)

Figure 3 presents the potential energy surfaces of Ac–l–Ala–Tzl(*4*–Me) (**1**) in three various environments; gas phase for isolated molecule, chloroform mimicking weakly polar inside of protein, and water as a natural solvent. Regardless of the simulated environment, five energy minima were found: β2, β, αL, αD, αR (Table 1).

**Table 1:** Selected torsion angles (°) of local minima for Ac-–l–Ala–Tzl(*4*–Me) (**1**), their relative energies (ΔE) in kcal/mol and population (*p*), optimised by M06–2X/6–311++G(d,p) method

Ac–l–Ala–Tzl(*4*–Me) (1)				
Conformer	φ	ψ	ΔE	*p* [%]
Gas phase				
β2	− 160.4	− 5.7	0.00	78.7
β	− 95.2	− 171.9	1.20	10.5
αR	− 82.8	− 106.0	1.22	10.1
αD	64.7	− 158.4	3.13	0.4
αL	56.4	36.7	3.38	0.3
Chloroform				
β	− 82.3	170.7	0.00	57.6
β2	− 159.2	− 8.13	0.48	25.9
αR	− 70.3	− 42.6	0.86	13.6
αD	64.1	− 154.1	1.98	2.1
αL	56.8	37.9	2.51	0.9
Water				
β	− 67.3	153.3	0.00	80.8
αR	− 65.3	− 38.9	1.10	12.8
αD	60.0	− 147.2	2.00	2.8
β2	− 159.1	− 8.6	2.10	2.4
αL	58.2	39.3	2.51	1.2

In the gas phase, the global minimum is occupied by the semi-extended conformation β2 (φ, ψ = − 160°, − 6°). Its population is quite high, around 79%. The stability of this conformation can be explained by the presence of intramolecular hydrogen bonds; N–H⋯N_TZL_ formed between the hydrogen atom of amide group and the nitrogen atom of thiazole ring as well as Cα–H⋯O hydrogen bond created by the hydrogen atom of α carbon atom and the oxygen atom of amide group. The parameters of intramolecular hydrogen bonds can be found in Table S1 in Supporting Information. Next in energy order are the conformation β (φ, ψ = − 95°, − 172°) and the right-handed helical αR (φ, ψ ~ 83°, 106°). They are expected to be adopted by 10% of the population each. Both conformations are stabilised by weaker Cα–H⋯O hydrogen bonds and electrostatic attractive interactions between the amide oxygen atom (NBO charge − 0.66) and thiazole sulfur atom (NBO charge + 0.40) (Tables S1 and S2). The remaining conformations αD (φ, ψ = 65°, − 158°) and αL (φ, ψ = 56°, 37°) have much higher energies (Δ*E* > 3 kcal/mol) and their populations are estimated on less than 0.5%. Their presence on the maps can be explained mainly by electrostatic interactions.

The increase of polarity of environment results in a change of energy order of conformations so that the structure β becomes the lowest one. Moreover, a decrease in energy gaps between the conformations is observed. This is because the conformations higher in energy for isolated molecule, αD, and αL, gain stability from interactions with polar solvent; whereas, the conformations β2, β, and αR still have functional group involved in internal interactions.

The potential energy surface is relatively flat in the regions surrounding three the lowest in energy conformations, β2, β, and αR. This indicates that considerable conformational changes can be made at a relatively low energy cost. In fact, conformers β and αR undergo significant geometrical changes, up to 30° and even 70°, respectively, in case of value of torsion angle ψ when the environment is changed from gas phase to water. In contrast, the geometry of conformations β2, αD, and αL seems to be independent of polarity of studied environments. The rigidity of conformation β2 seems to result from the presence of the internal N–H⋯N_TZL_ hydrogen bond. It is still maintained in a more polar environment; however, the amide N–H group is not involved in an intermolecular interaction, and thus its relative energy increases. The rigidity of conformations αD and αL does not result from any stable internal stabilising forces, but rather from steric repulsion. This indicates that with the increase of polarity of environment, stability is gained rather from intermolecular than intramolecular interactions.

### Thiazole–dehydroalanine

**Fig. 4:**
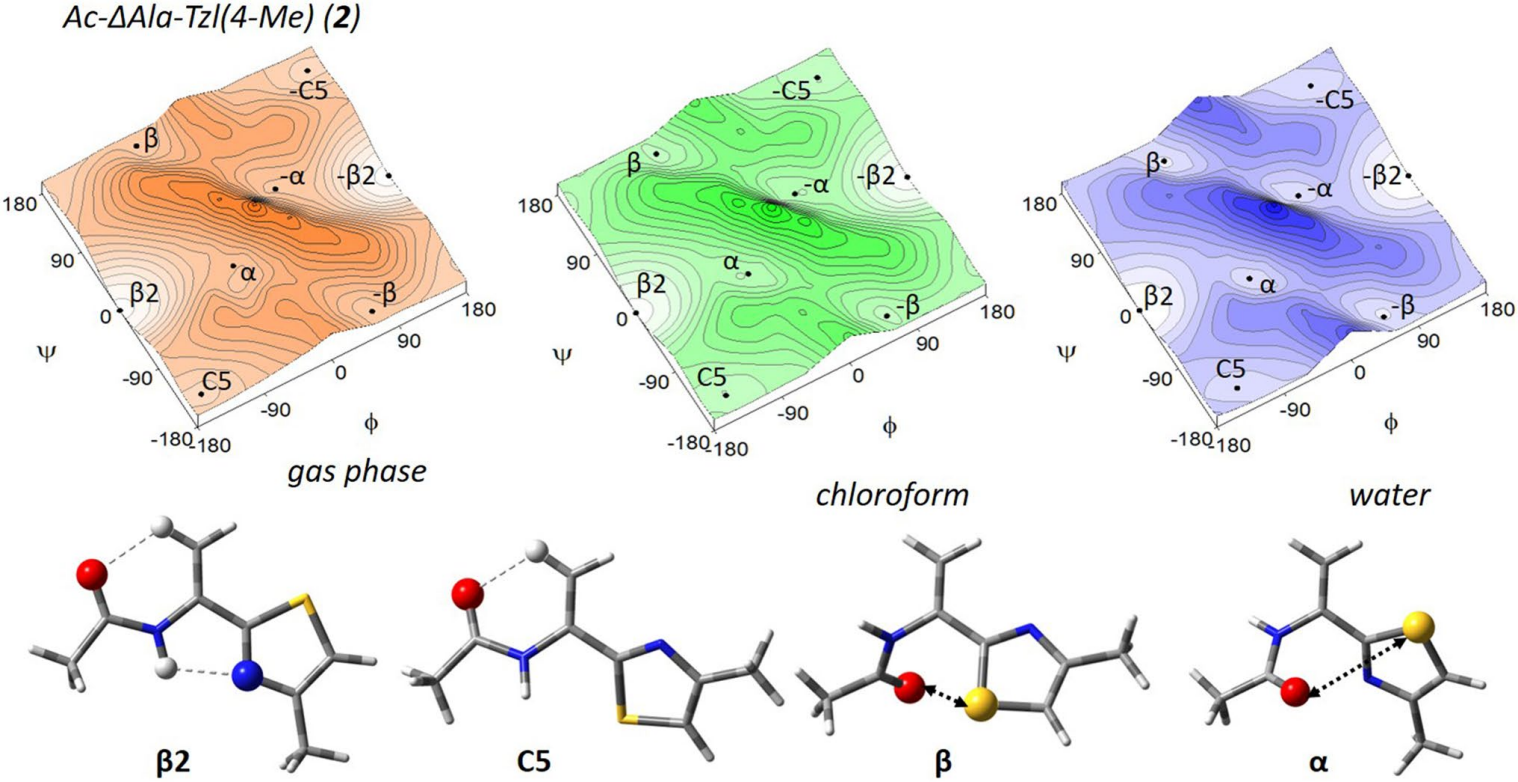
The potential energy surfaces E = f(φ,ψ) for Ac–∆Ala–Tzl(4–Me) (2) calculated by M06-2X/6–311++G(d,p) method in various environment. Energy contours are plotted every 1 kcal/mol. Conformations optimised in chloroform with most important electrostatic interactions (◄⋯►) and hydrogen bond (⋯)

The conformational maps for Ac–ΔAla–Tzl(*4*–Me) (**2**) show that for this residue four pairs of potential energy minima can be possible (Fig. 4). Due to the lack of chirality of the carbon atom α the maps are symmetric. Considering the left side of maps four minima can be found, β2, C5, β, and α, but their analogues with the same energy but opposite sign of torsion angles are present on the right side (Table 2). The number and types of conformations do not change, regardless of the simulated environment.

**Table 2:** Selected torsion angles (°) of local minima for Ac–ΔAla–Tzl(*4*–Me) (2), their relative energies (ΔE) in kcal/mol, and population (*p*), optimised by M06–2X/6–311++G(d,p) method

Ac–ΔAla–Tzl(*4*–Me) (2)				
Conformer	φ	ψ	ΔE	*p* [%]
Gas phase				
β2	− 180.0	0.0	0.00	99.94
C5	− 161.3	− 156.4	4.56	0.05
β	− 62.9	164.0	5.80	0.01
α	47.1	32.7	6.08	0.00
Chloroform				
β2	− 180.0	0.0	0.00	98.87
C5	− 150.5	− 156.0	3.09	0.55
β	− 61.1	159.1	3.30	0.39
α	49.8	32.4	3.72	0.19
Water				
β2	− 180.0	0.0	0.00	68.56
β	− 58.3	153.8	0.86	16.20
α	52.0	33.7	1.18	9.48
C5	− 137.9	− 153.5	1.47	5.77

Both in the gas phase and the studied solvents, the global minimum corresponds to the conformation β2 (φ,ψ ≈ 180°, 0°), which is stabilszed mainly by the intramolecular N–H⋯N_TZL_ hydrogen bond, created between the amide group and thiazole nitrogen atom. There is also the Cβ–H⋯O interaction. The values of torsion angles φ and ψ indicate the flatness of structure, due to the presence of α,β-double bond neighbouring with the amide group and thiazole ring so that the conformation β2 gains stability from the cross-conjugate π-electron system. The remaining conformation C5 (φ,ψ ≈ − 161°, − 156°), β (φ,ψ ≈ − 63°, − 164°), and α (φ,ψ ≈ 47°, 33°) are stabilised only by the Cβ–H⋯O (C5), and electrostatic interactions (β and α). The values of torsion angles indicate that for these conformations the π-electron conjugation is insignificant. This explains their high relative energy.

The results of calculations show that also in the polar environment the conformer β2 is also the most stable. Its population is very high in gas phase and chloroform, around 99%, and still prevails in water (69%). The geometry does not change, which indicates stability gained from intramolecular forces. The relative energies of the remaining conformers, C5, β, and α, are still high in a weakly polar chloroform, but considerably decrease in water. On the other hand, the geometry of conformations is generally maintained, except for the conformer C5, where the angle φ changes by about 23° when switching from gas to water. The presented results indicate that the environment does not influence the geometry of conformations of thiazole–dehydroalanine residue, but it does the relative energy through the interactions with the solvent, which stabilised all conformers.

The conformation C5 is not available for the saturated analogue (**1**). Comparing the shape of conformational maps for molecules (**1**) and (**2**), it seems that minima are better defined for (**2**). The presence of double bond in the side chain gives the ability to create additional stabilising force, the π-electron conjugation, which considerably increases the stability of flat conformers: β2 and C5. This causes, that the minima found for Ac–ΔAla–Tzl(*4*–Me) are more stable and have less conformational freedom than those found for Ac–l–Ala–Tzl(*4*–Me). However, in water, the energy differences between conformations are blurring.

### Thiazole–(*Z*)–dehydrobutyrine

**Fig. 5:**
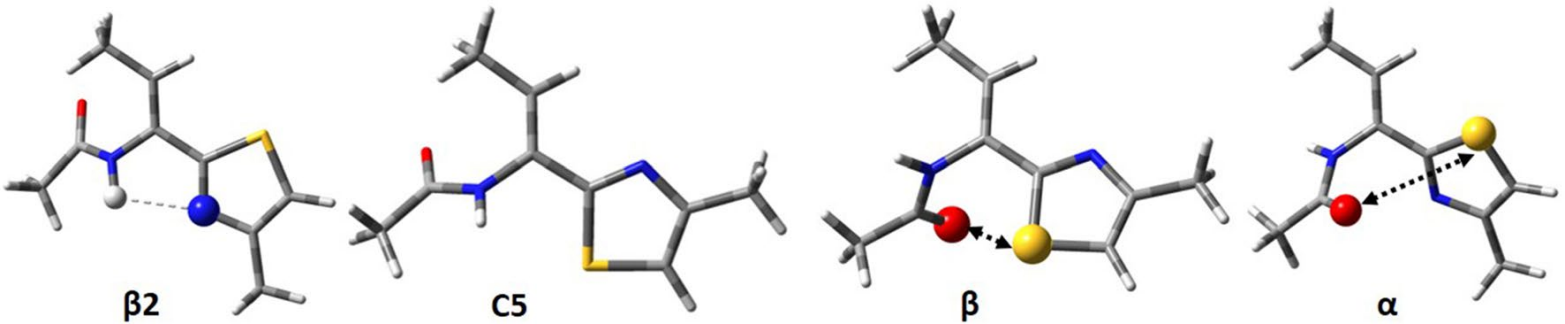
Conformers for Ac–(Z)–ΔAbu–Tzl(4–Me) (3) calculated by M06–2X/6–311++G(d,p) method in chloroform with most important electrostatic interactions (◄⋯►) and hydrogen bond (⋯)

Figure 5 and Table 3 present the four conformers found for Ac–(Z)–ΔAbu–Tzl(*4*–Me) (**3**): β2 (φ,ψ ≈ − 126°, − 4°), C5 (φ,ψ ≈ − 123°, 164°), β (φ,ψ ≈ − 69°, 169°), and α (φ,ψ ≈ 56°, 22°). The number and type of conformations are the same as in the case of analogue (**2**). Also, the energy order of conformations is the same both for isolated molecule and in a weakly polar environment. However, difference occurs in case of the conformers β2 and C5, where the value of torsion angle φ is about − 120° due to the steric hindrance imposed by the methyl group in position *Z* of the side chain. This causes that Cβ–H⋯O H-bond is absent and N–H⋯N hydrogen bond and π-electron conjugation are distorted. In consequence, the conformation β2 is less stable what diminishes the energy gap between the conformations so that the conformation β2 is less dominant. In gas phase its population reaches 90%, but in chloroform it diminishes to 55%, and in water further decreases to 22%. In the water environment, the global minimum is changed, the lowest in energy is the conformation C5. The population of the first three conformations occurs with a similar probability of around 30%. It should be also noticed that the energy differences are within the error bar of method and do not exceed 1 kcal/mol. Therefore, in this case, none of the conformation is favoured.

**Table 3:** Selected torsion angles (°) of local minima for Ac–(Z)–ΔAbu–Tzl(*4*–Me) (**3**), their relative energies (ΔE) in kcal/mol and population (*p*), optimised by M06-2X/6–311++G(d,p) method

Ac–(Z)–ΔAbu–Tzl(4–Me) (3)					
Conformer	φ	ψ		ΔE	*p* [%]
Gas Phase					
β2	− 125.8	− 3.5		0.00	90.3
C5	− 122.8	− 164.1		1.37	9.0
β	− 69.2	169.4		3.12	0.5
α	56.3	22.2		3.56	0.2
Chloroform					
β2	− 124.5	2.8		0.00	55.4
C5	122.3	161.3		0.34	31.2
β	− 66.4	163.5		1.07	9.3
α	52.3	29.4		1.54	4.2
Water					
C5	118.9	160.2		0.00	35.2
β	− 62.5	156.6		0.10	29.7
β2	− 122.3	16.4		0.27	22.5
α	54.7	32.5		0.61	12.6

### Thiazole–dehydrophenylalanine

**Fig. 6:**
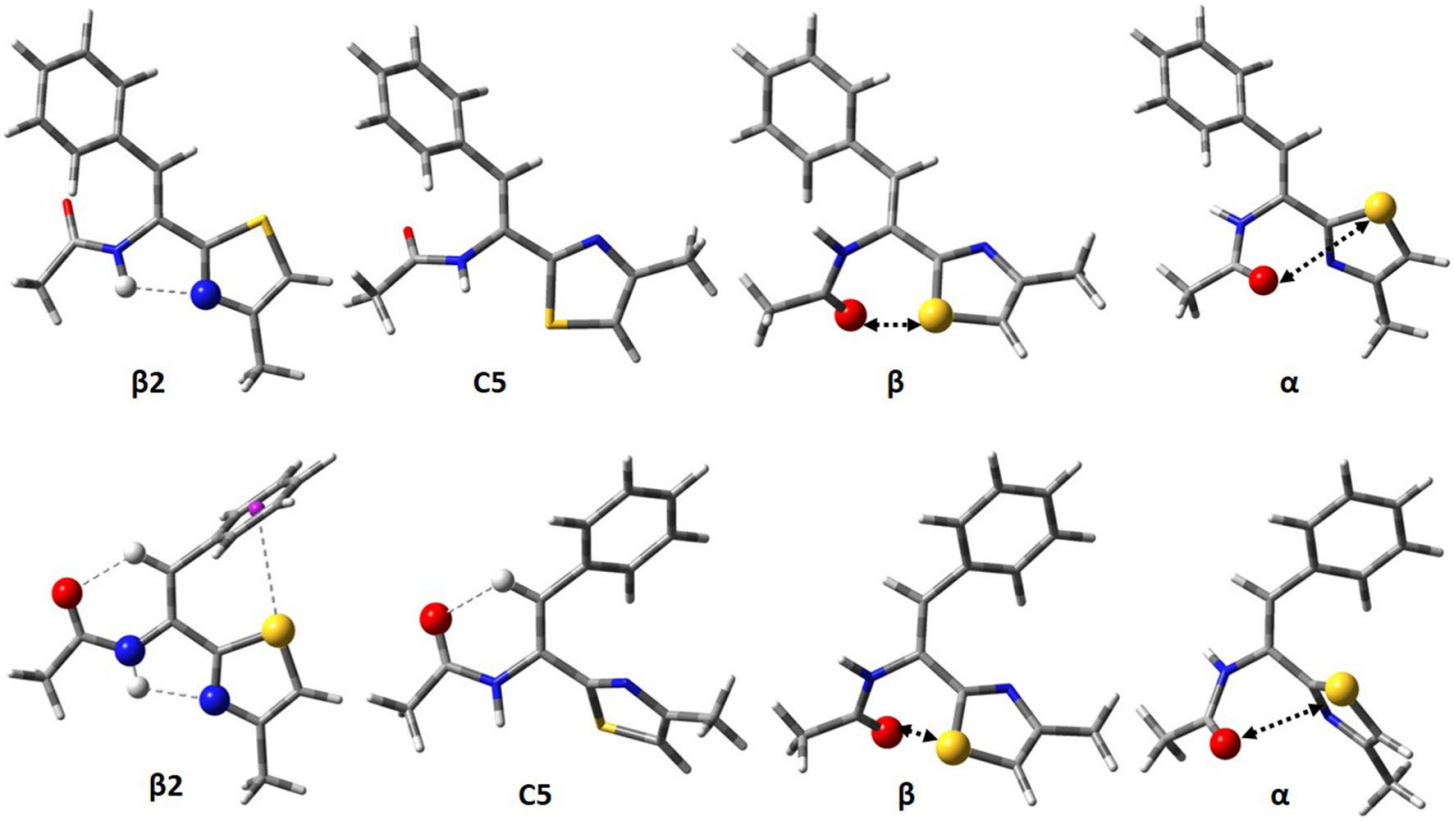
Conformers for Ac–(Z)–ΔPhe–Tzl(4–Me) (4) and Ac–(E)–ΔPhe–Tzl(4–Me) (5) calculated by M06–2X/6–311++G(d,p) method in chloroform with most important electrostatic interactions (◄⋯►) and hydrogen bond (⋯)

Both geometric isomers of thiazole–dehydrophenylalanine can adopt, regardless of the environment, four different conformations: β2, β, C5, and α (Fig. 6, Table 4). The geometry of conformations for Ac–(Z)–ΔPhe–Tzl(*4*–Me) (**4**): β2 (φ,ψ ≈ − 129°, 0°), β (φ,ψ ≈ − 69°, 167°), α (φ,ψ ≈ 48°, 32°), and C5 (φ,ψ ≈ − 123°, 165°) is very similar to the analogue (**3**). However, the energy difference (ΔE) between the conformations is considerably smaller. Although for the isolated molecule the conformation β2 prevails, in a polar environment a tendency towards the conformation β is observed. Nevertheless, ΔE is below 1 kcal/mol, so the conformational equilibrium is predicted. It seems that the phenyl ring imposes greater steric hindrance than the methyl group in the case of (**3**).

**Table 4:** Selected torsion angles (°) of local minima for Ac-(*Z*)–ΔPhe–Tzl(*4*–Me) (**4**) and Ac–(*E*)–ΔPhe–Tzl(*4*–Me) (**5**), their relative energies (ΔE) in kcal/mol and population (*p*), optimised by M06–2X/6–311++G(d,p) method

Conformer	φ	ψ	τ	χ	ΔE	*p* [%]
Ac–(Z)–ΔPhe–Tzl(4–Me) (4)						
Gas phase						
β2	− 129.2	0.1	8.7	23.8	0.00	66.1
β	− 68.6	167.3	− 7.2	− 41.7	0.62	23.2
α	48.0	31.5	7.2	38.5	1.42	6.0
C5	− 123.3	− 165.4	6.8	33.1	1.58	4.6
Chloroform						
β	− 63.6	161.6	− 6.6	− 34.6	0.00	42.8
β2	− 127.4	5.9	9.0	21.4	0.34	24.1
α	50.8	30.4	7.1	35.1	0.55	17.1
C5	− 121.7	− 164.7	6.5	32.7	0.59	16.0
Water						
β	− 64.5	157.8	− 6.4	− 30.5	0.00	47.5
C5	− 122.2	− 161.4	5.9	26.6	0.40	24.2
α	57.2	29.4	7.0	33.2	0.71	14.4
β2	− 127.0	16.9	8.3	20.3	0.73	13.8
Ac–(E)–ΔPhe–Tzl(4–Me) (5)						
Gas phase						
β2	− 179.3	0.1	− 179.9	− 91.2	0.00	99.9
α	40.6	48.7	− 168.2	38.4	4.66	0.0
C5	− 161.2	− 130.7	− 173.5	40.1	4.73	0.0
β	− 56.6	146.1	169.9	47.4	7.00	0.0
Chloroform						
β2	− 179.8	− 1.3	179.5	93.9	0.00	93.7
α	41.3	48.9	− 168.2	38.8	1.86	4.1
C5	− 146.5	− 134.7	− 171.7	39.3	2.26	2.1
β	− 54.3	141.5	169.5	49.9	4.18	0.1
Water						
α	44.9	46.7	− 168.9	40.1	0.00	39.9
β	− 48.5	134.3	168.9	− 35.8	0.10	33.8
β2	− 149.7	48.2	− 172.7	39.9	0.48	17.8
C5	− 139.5	− 131.4	− 171.3	36.8	0.93	8.4

Analysis of conformations of the isomer *E*, Ac–(*E*)–ΔPhe–Tzl(4–Me) (**5**): β2 (φ,ψ ≈ − 179°, 0°), α (φ,ψ ≈ 41°, 49°), C5 (φ,ψ ≈ − 161°, − 131°), and β (φ,ψ ≈ − 57°, 146°), shows differences in geometry and relative energy between the isomers. The conformation β2 is flat and resembles that for the thiazole–dehydroalanine (**2**). The parameters of N–H⋯N hydrogen bond are better than for the analogue *Z* (**4**) (Table S1). Additionally, an interaction between the aromatic ring and sulfur atom can be considered. The phenyl group in position *E* does not impose steric hindrance on the N-terminal amide group so that the value of torsion angle φ is close to 180° and the π-electron conjugation between N-terminal amide group and Cα = Cβ double bond should be present. In results, the energy of the conformation β2 for isolated molecules and even for the weakly polar environment has considerably low energy, so that the conformation β2 gathers almost the whole population of molecules. In a more polar water environment, sterically more open conformations α and β prevail, due to better interaction with solvent, and the position of substituent in the side chain seems does not influence their geometry. In contrast, in the conformation C5 the phenyl ring imposes a steric hindrance, thus the value of torsion angle ψ is the lowest amongst the studied residues.

### Solid-state conformations from CSD

**Fig. 7:**
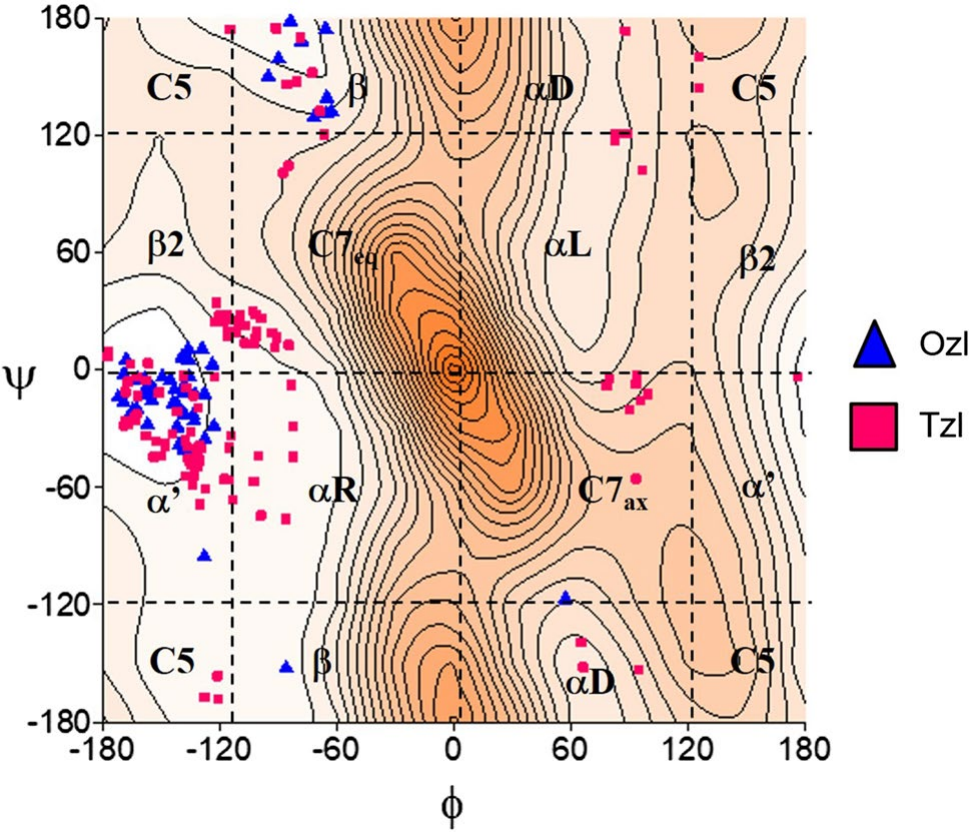
The potential energy surface, E = f(φ,ψ), for Ac–l–Ala–Tzl(4–Me) calculated by M06–2X/6–311++G(d,p) method in vacuo with solid-state crystal structure conformations of thiazole–amino acid residues (pink square) and oxazole–amino acid residues (blue triangular) retrieved from the Cambridge Structural Database. Energy contours are plotted every 1 kcal/mol

The solid-state crystal structure conformations of thiazole–amino acid residues, as well as oxazole–amino acid residues, were retrieved from the Cambridge Structural Database (Groom et al. 2016) and presented on the potential energy surface calculated for l–Ala–Tzl (**1**) (Fig. 7).

The amino acid residues found adjacent to the triazole ring are mainly *valine-* (Doi et al. 2001; McDonald et al. 1992; Todorova et al. 1995; Haberhauer et al. 2008; Asano et al. 2001, 2002a, 2002b, 2003, 2005; Asano and Doi 2004; Ishida et al. 1992; In et al. 1994; You and Kelly 2005; Bertram et al. 2003), *phenylalanine-* (Mali et al. 2012; Pettit et al. 1994, 2004), *glycine-* (Li et al. 2013; Bernhardt et al. 2002; Seiser et al. 2008; Kaiser et al. 2000; Stezowski et al. 1987) and *alanine-* (Schmitz et al. 1989; Vollbrecht et al. 2002; Zhou et al. 2012; Breydo et al. 2002) and other (Fletton et al. 1983; Nicolaou et al. 2003; Merino et al. 1998; Dondoni et al. 1995; Marsh 1997). Amongst 139 thiazole–amino acid residues, 81% can be found in the region of the conformation β2, and 10% adopt the conformation β. There are also structures, which represent the conformations αR, αL, and αD, although their population is considerably smaller. It should be noted that the residues occur in macrocyclic peptides, thus the geometrical strains in those macrocycles can have some impact on the adopted conformation. Nevertheless, it can be confirmed that the calculation relatively good predicts the conformations adopted by the thiazole–amino acid residues and also show that the side chain is not the leading feature that causes conformational propensity.

### Comparison to oxazole–amino acid

In our previous studies, we have analysed the conformational properties of oxazole–amino acid for analogous residues (Siodłak et al. 2014a; Staś et al. 2016b). Comparison of the relative energy of oxazole– and thiazole–amino acid conformers with the same side chain (Fig. 3S) shows similar conformational profiles; the type of conformations and energy order. Nevertheless, some differences can be seen. Thiazole–alanine (Ala–Tzl), and by analogy, other saturated side chain residues, have more available low-energy conformations (β2 and β) than oxazole–alanine (Ala–Ozl), at least in a low polar environment. In contrast, thiazole–dehydroalanine (ΔAla-Tzl) shows greater conformational restriction than oxazole analogue, and it seems that it has a much greater tendency to adopt solely the conformation β2. A similar effect can be seen for thiazole–dehydrophenylalanine (ΔPhe–Tzl), both isomers *Z* and *E*, for isolated molecules. Thiazole–dehydrobutyrine (*Z*–ΔAbu–Tzl) seems to have more conformational freedom, regardless of the mimicking environment.

### Interaction between a water molecule and thiazole / oxazole ring

**Fig. 8:**
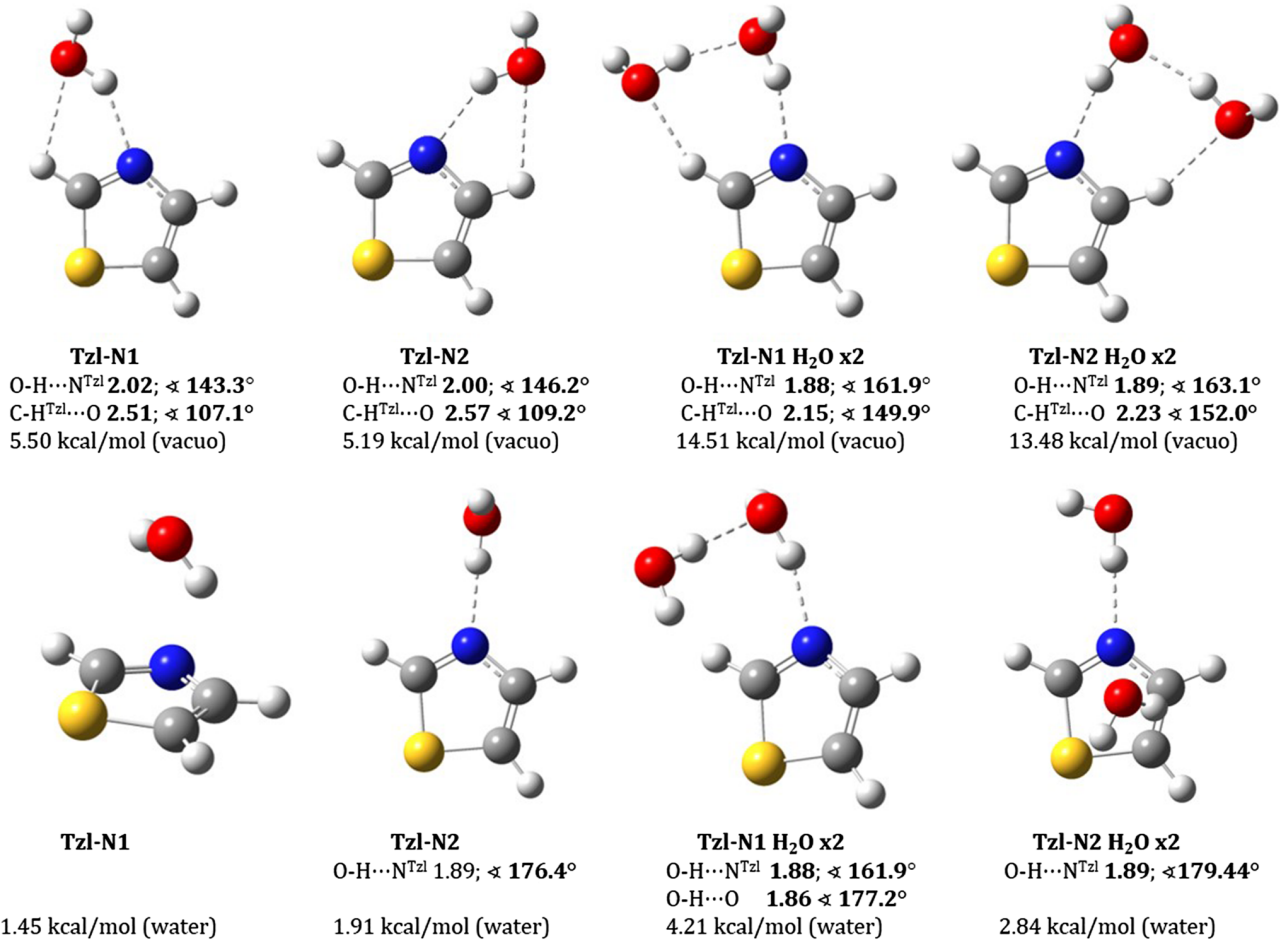
Water–thiazole ring complexes with interaction energies calculated by M06–2X/6–311++G(d,p) method

In order to gain further information about the role of thiazole ring in the creation of intra- and intermolecular interactions, crucial for existence and energy order of thiazole–amino acid conformations, the interaction of water molecule with thiazole ring was simulated by MD. According to the simulations, in the first solvation shell of thiazole ring (in the distance of 2.5 Å) are present from none to three water molecules, in case of oxazole ring it is from none to four. Percentage analysis for 200 snapshots are shown in the Table S3. The interaction of thiazole ring with one water molecule it is always from the nitrogen atom side. Examples of complexes optimised by DFT in vacuo and water are in Fig. 8. The estimated strength of hydrogen bond between one water molecule and thiazole ring is about 5 kcal/mol (Tzl–N1 and Tzl–N2) for isolated complexes (Fig. 8). If the water environment is considered, the energy was estimated at 2 kcal/mol. The analogous calculations for the oxazole ring still indicate some energy profit (Figure S2). The simulation shows that despite structural similarity, thiazole ring moiety will have a different influence on conformational properties than oxazole analogue. It also explains why a nitrogen atom in the thiazole is a preferred site for metal chelation (Le et al. 2010). The sulfur atom contributes with its lone pair to an electronic sextet; whereas, the nitrogen atom has free lone pair ready for interactions with other atoms. In the C–S bond is created σ-hole, due to sulfur low-lying σ* orbitals. It causes that this atom has positive electrostatic potential in the thiazole ring.

## Conclusions

Conformational analysis of selected thiazole–amino acid residues, most commonly found in nature, indicates that the combination of two structural motifs, amino acid side chain and thiazole ring gives unique properties of such residues.

The characteristic feature is the low-energy conformation β2, unusual for standard amino acid residues. This conformation is stabilised by an intramolecular hydrogen bond N–H⋯N_TZL_, between the N-terminal N–H amide group and the nitrogen atom of the thiazole ring. Analysis of thiazole–alanine (**1**) supported by the conformations of the solid-state crystal structures retrieved from the Cambridge Structural Database shows that this unique semi-extended conformation β2 (ϕ, ψ ~ − 160°, 8°) is characteristic also for other thiazole–amino acid residues. The population of conformation β2 is considerable at least in a low polar environment.

The thiazole–dehydroamino acids (**2–5**) have different conformational profiles due to the presence of Cα = Cβ double bond and the lack of chirality. Nevertheless, the conformation β2 seems to be even more stable due to π-electron cross-conjugation. It is heavily populated for thiazole–dehydrobutyrine (**2**) (ϕ, ψ ~ − 125°, 5°) or even predominate for naturally occurring thiazole–dehydroalanine (**1**) (ϕ, ψ ~ − 180°, 0°), regardless of the studied environment. Analysis of the *Z* and *E* geometric isomers of thiazole–dehydrophenylalanine (**4** and **5**) indicates that the position of side chain is important, pointing to a potential conformational switch.

The properties of thiazole ring and the presence of sulfur atom are also far-reaching. The positive electrostatic potential of the sulfur atom creates possible intramolecular electrostatic interactions which, although relatively weak, results in some conformational differences as compared to oxazole–amino acids.

Our studies have been inspired by naturally occurring, highly structurally modified peptides produced by microorganisms that differentiated their bioactivities. We believe that our findings may be useful in understanding the bioactive conformations of these natural peptides containing the structural units studied. The unique properties of such non-standard amino acid residues should result in increasing interest and point out the potential application in drug design.

## Supplementary Information

Below is the link to the electronic supplementary material.

Supplementary file1 (DOCX 882 KB)
