# Supplementary material for: Thiazole–amino acids: influence of thiazole ring on conformational properties of amino acid residues
Source: Amino Acids. 2021 Apr 10;53(5):673–86. doi: 10.1007/s00726-021-02974-0 (PMC8128816; doi:10.1007/s00726-021-02974-0)
Supplement: Supplementary file 1 — Supplementary file1 (DOCX 882 KB) [file 726_2021_2974_MOESM1_ESM.docx]

# SUPPORTING INFORMATION

# Thiazole Amino Acids. Influence of Thiazole Ring on Conformational Properties of Amino Acid Residues

Monika Staś*^1,2^, Małgorzata A. Broda^1^, Dawid Siodłak*^1^

*^1^Faculty of Chemistry, University of Opole, Opole 45-052, Poland*

*^2^Institute of Organic Chemistry and Biochemistry of the Czech Academy of Science, Flemingovo Náměstí 2, 166 10 Praha 6, Czech Republic*

e-mail: [mstas@uni.opole.pl](mailto:mstas@uni.opole.pl), [plgmonika691@gmail.com](mailto:plgmonika691@gmail.com)

**Table 1S.** Structural parameters of internal hydrogen bond X–H···A (X = N, C; A = O,N) according to Ref. [1] in various environment for molecules **1-5**, the full optimization was carried out by M06-2X/6-311++G(d,p) method.

|  | **Ac-L-Ala-Tzl(*4*-Me) (1)** | | | | | | | | | | | | | | |
| --- | --- | --- | --- | --- | --- | --- | --- | --- | --- | --- | --- | --- | --- | --- | --- |
|  | **β2** | | | **αR** | | | **β** | | | **αL** | | | **αD** | | |
|  | *vacuo* | *chloroform* | *water* | *vacuo* | *chloroform* | *water* | *vacuo* | *chloroform* | *water* | *vacuo* | *chloroform* | *water* | *vacuo* | *chloroform* | *water* |
| **N^N^-H···N^C^** |  |  |  |  |  |  |  |  |  |  |  |  |  |  |  |
| *r* H···N | 2.22 | 2.27 |  |  |  |  |  |  |  |  |  |  |  |  |  |
| *r* N···N | 2.70 | 2.71 |  |  |  |  |  |  |  |  |  |  |  |  |  |
| ∠N-H···N | 106.9 | 105.0 |  |  |  |  |  |  |  |  |  |  |  |  |  |
| ∠ H···N-C | 83.4 | 83.0 |  |  |  |  |  |  |  |  |  |  |  |  |  |
| **C^α^-H··· O^N^** |  |  |  |  |  |  |  |  |  |  |  |  |  |  |  |
| *r* H···O | 2.55 | 2.55 | 2.55 | 2.42 | 2.56 | 2.64 | 2.38 | 2.52 | 2.64 |  |  |  |  |  |  |
| *r* C···O | 2.76 | 2.77 | 2.78 | 2.77 | 2.76 | 2.77 | 2.80 | 2.78 | 2.77 |  |  |  |  |  |  |
| ∠C-H···O | 89.0 | 89.9 | 90.3 | 97.1 | 89.0 | 84.8 | 101.3 | 91.9 | 84.8 |  |  |  |  |  |  |
| ∠C=O···H | 79.2 | 79.4 | 79.4 | 82.1 | 80.4 | 78.5 | 82.6 | 80.5 | 78.6 |  |  |  |  |  |  |
| **C^β^-H···O^N^** |  |  |  |  |  |  |  |  |  |  |  |  |  |  |  |
| *r* H···O |  |  |  |  |  |  |  |  |  | 2.44 | 2.52 | 2.61 | 2.46 | 2.52 | 2.60 |
| *r* C···O |  |  |  |  |  |  |  |  |  | 3.03 | 3.09 | 3.16 | 3.06 | 3.09 | 3.14 |
| ∠C-H···O |  |  |  |  |  |  |  |  |  | 112.5 | 111.4 | 110.1 | 113.5 | 111.3 | 110.0 |
| ∠C=O···H |  |  |  |  |  |  |  |  |  | 101.2 | 98.2 | 94.5 | 98.2 | 97.4 | 95.4 |

|  | **Ac-ΔAla-Tzl(*4*-Me) (2)** | | | | | | **Ac-(*Z*)-ΔAbu-Tzl(4-Me) (3)** | | | **Ac-(*Z*)-ΔPhe-Tzl(4-Me) (4)** | | | **Ac-(*E*)-ΔPhe-Tzl(4-Me) (5)** | | | | | |
| --- | --- | --- | --- | --- | --- | --- | --- | --- | --- | --- | --- | --- | --- | --- | --- | --- | --- | --- |
|  | **β2** | | | **C5** | | | **β2** | | | **β2** | | | **β2** | | | **C5** | | |
|  | *vacuo* | *chloroform* | *water* | *vacuo* | *chloroform* | *water* | *vacuo* | *chloroform* | *water* | *vacuo* | *chloroform* | *water* | *vacuo* | *chloroform* | *water* | *vacuo* | *chloroform* | *water* |
| **N^N^-H···N^C^** |  |  |  |  |  |  |  |  |  |  |  |  |  |  |  |  |  |  |
| *r* H···N | 2.17 | 2.19 | 2.20 |  |  |  | 2.30 | 2.44 | 2.66 | 2.30 | 2.35 | 2.55 | 2.09 | 2.10 | 2.73 |  |  |  |
| *r* N···N | 2.68 | 2.69 | 2.70 |  |  |  | 2.72 | 2.75 | 2.80 | 2.70 | 2.72 | 2.78 | 2.63 | 2.64 | 2.92 |  |  |  |
| ∠N-H···N | 109.7 | 108.7 | 108.4 |  |  |  | 103.9 | 97.0 | 87.4 | 106.4 | 100.8 | 92.2 | 111.4 | 110.7 | 90.5 |  |  |  |
| ∠ H···N-C | 83.0 | 82.8 | 82.6 |  |  |  | 81.7 | 79.6 | 74.0 | 82.0 | 80.1 | 75.3 | 84.9 | 84.8 | 65.2 |  |  |  |
| **C^β^-H···O^N^** |  |  |  |  |  |  |  |  |  |  |  |  |  |  |  |  |  |  |
| *r* H···O | 2.24 | 2.25 | 2.27 | 2.31 | 2.41 | 2.58 |  |  |  |  |  |  | 2.14 | 2.15 | 2.39 | 2.25 | 2.42 | 2.54 |
| *r* C···O | 2.87 | 2.88 | 2.89 | 2.89 | 2.92 | 2.96 |  |  |  |  |  |  | 2.87 | 2.88 | 2.91 | 2.90 | 2.93 | 3.04 |
| ∠C-H···O | 115.6 | 115.2 | 114.4 | 112.2 | 107.2 | 100.1 |  |  |  |  |  |  | 122.2 | 122.2 | 107.8 | 115.9 | 107.2 | 101.6 |
| ∠C=O···H | 105.2 | 105.3 | 105.2 | 102.2 | 98.4 | 92.6 |  |  |  |  |  |  | 103.3 | 103.3 | 95.9 | 100.7 | 94.5 | 90.5 |
| ^N. C^ denote the N-terminal carbonyl group and C-terminal in thiazole ring | | | | | | | | | | | | | | | | | | |

**Table S2.** The NBO charges of selected atoms (according to drawings) for the studied compounds in all conformations calculated in gas phase by M06-2X/6-311++G(d,p) level of theory.


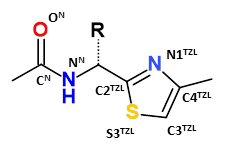


|  | **O^N^** | **C^N^** | **N^N^** | **N1^Tzl^** | **C2^Tzl^** | **S3^Tzl^** | **C4^Tzl^** | **C5^Tzl^** |
| --- | --- | --- | --- | --- | --- | --- | --- | --- |
| **Ac-L-Ala-Tzl(4-Me) (1)** | | | | | | | | |
| **β2** | -0.66 | 0.70 | -0.67 | -0.53 | 0.10 | 0.40 | -0.42 | 0.13 |
| **αR** | -0.65 | 0.70 | -0.66 | -0.51 | 0.07 | 0.44 | -0.43 | 0.12 |
| **β** | -0.63 | 0.70 | -0.68 | -0.51 | 0.09 | 0.40 | -0.43 | 0.13 |
| **αL** | -0.63 | 0.71 | -0.67 | -052 | 0.09 | 0.41 | -0.43 | 0.13 |
| **αD** | -0.63 | 0.70 | -0.68 | -0.50 | 0.10 | 0.39 | -0.43 | 0.13 |
| **Ac-ΔAla-Tzl(4-Me) (2)** | | | | | | | | |
| **β2** | -0.62 | 0.71 | -0.66 | -0.52 | 0.06 | 0.42 | -0.42 | 0.14 |
| **C5** | -0.61 | 0.71 | -0.66 | -0.48 | 0.06 | 0.38 | -0.43 | 0.14 |
| **β** | -0.60 | 0.70 | -0.69 | -0.49 | 0.06 | 0.42 | -0.43 | 0.14 |
| **α** | -0.61 | 0.71 | -0.67 | -0.48 | 0.06 | 0.42 | -0.43 | 0.14 |
| **Ac-(*Z*)-ΔAbu-Tzl(4-Me) (3)** | | | | | | | | |
| **β2** | -0.62 | 0.71 | -0.67 | -0.52 | 0.07 | 0.40 | -0.42 | 0.13 |
| **C5** | -0.62 | 0.70 | -0.68 | -0.49 | 0.07 | 0.39 | -0.43 | 0.14 |
| **β** | -0.61 | 0.70 | -0.70 | -0.50 | 0.07 | 0.41 | -0.43 | 0.14 |
| **α** | -0.61 | 0.72 | -0.69 | -0.50 | 0.07 | 0.41 | -0.42 | 0.13 |
| **Ac-(*Z*)-ΔPhe-Tzl(4-Me) (4)** | | | | | | | | |
| **β2** | -0.59 | 0.71 | -0.68 | -0.53 | 0.06 | 0.41 | -0.42 | 0.14 |
| **C5** | -0.60 | 0.71 | -0.69 | -0.49 | 0.06 | 0.39 | -0.43 | 0.14 |
| **β** | -0.60 | 0.70 | -0.69 | -0.49 | 0.06 | 0.43 | -0.43 | 0.14 |
| **α** | -0.61 | 0.71 | -0.67 | -0.48 | 0.06 | 0.42 | -0.43 | 0.14 |
| **Ac-(*E*)-ΔPhe-Tzl(4-Me) (4)** | | | | | | | | |
| **β2** | -0.63 | 0.71 | -0.65 | -0.53 | 0.05 | 0.47 | -0.43 | 0.14 |
| **C5** | -0.61 | 0.70 | -0.65 | -0.46 | 0.05 | 0.39 | -0.43 | 0.14 |
| **β** | -0.60 | 0.70 | -0.68 | -0.49 | 0.08 | 0.41 | -0.42 | 0.14 |
| **α** | -0.61 | 0.71 | -0.66 | -0.49 | 0.05 | 0.46 | -0.42 | 0.13 |


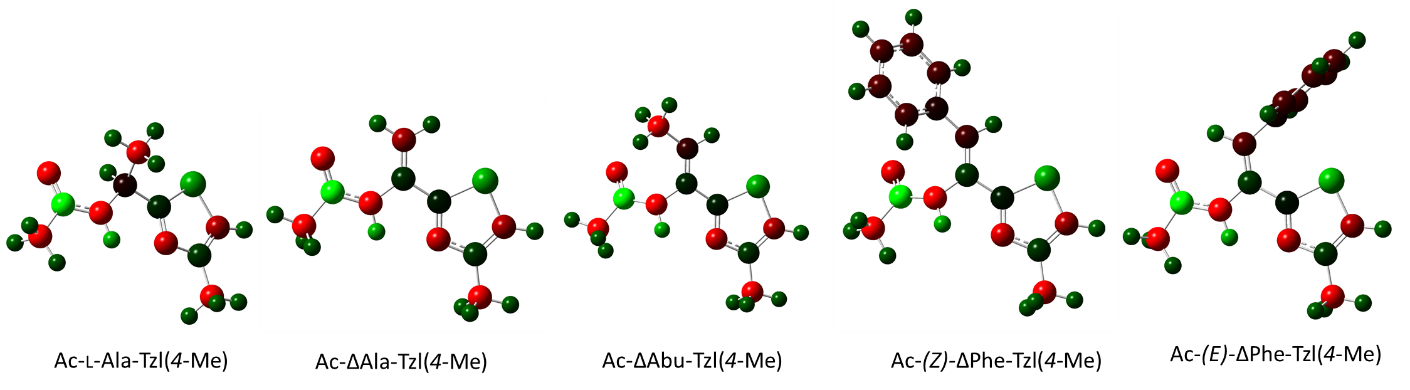

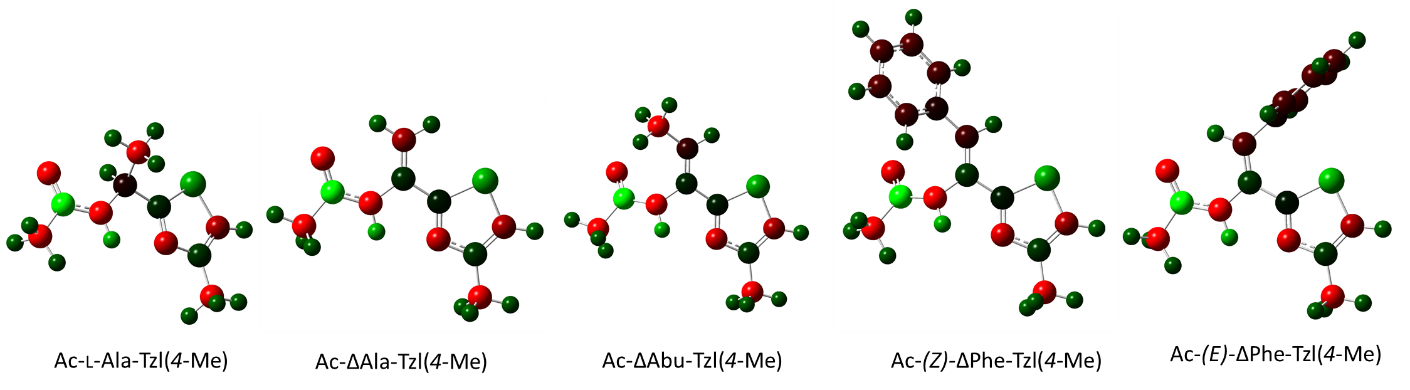

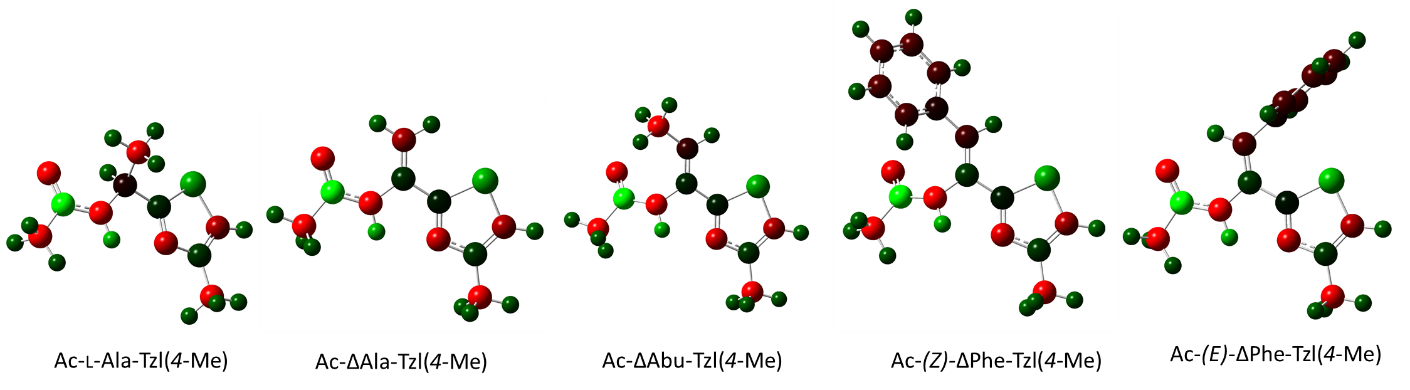


**Figure S1.** Visual representation of NBO charges for the studied compounds in conformation β2 calculated in gas phase by M06-2X/6-311++G(d,p) level of theory. The colures range from green – (positive charge) to red (negative charge).


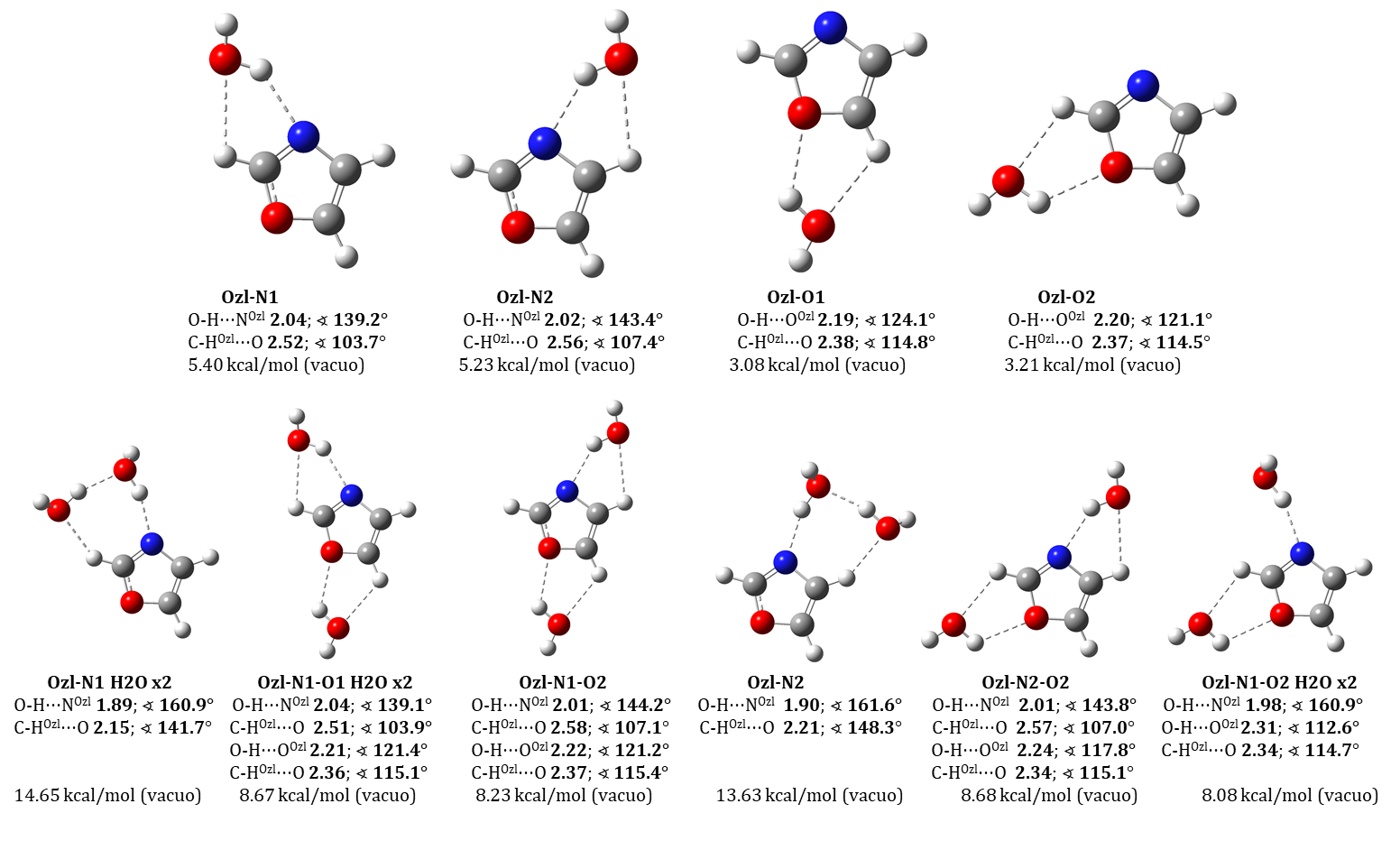

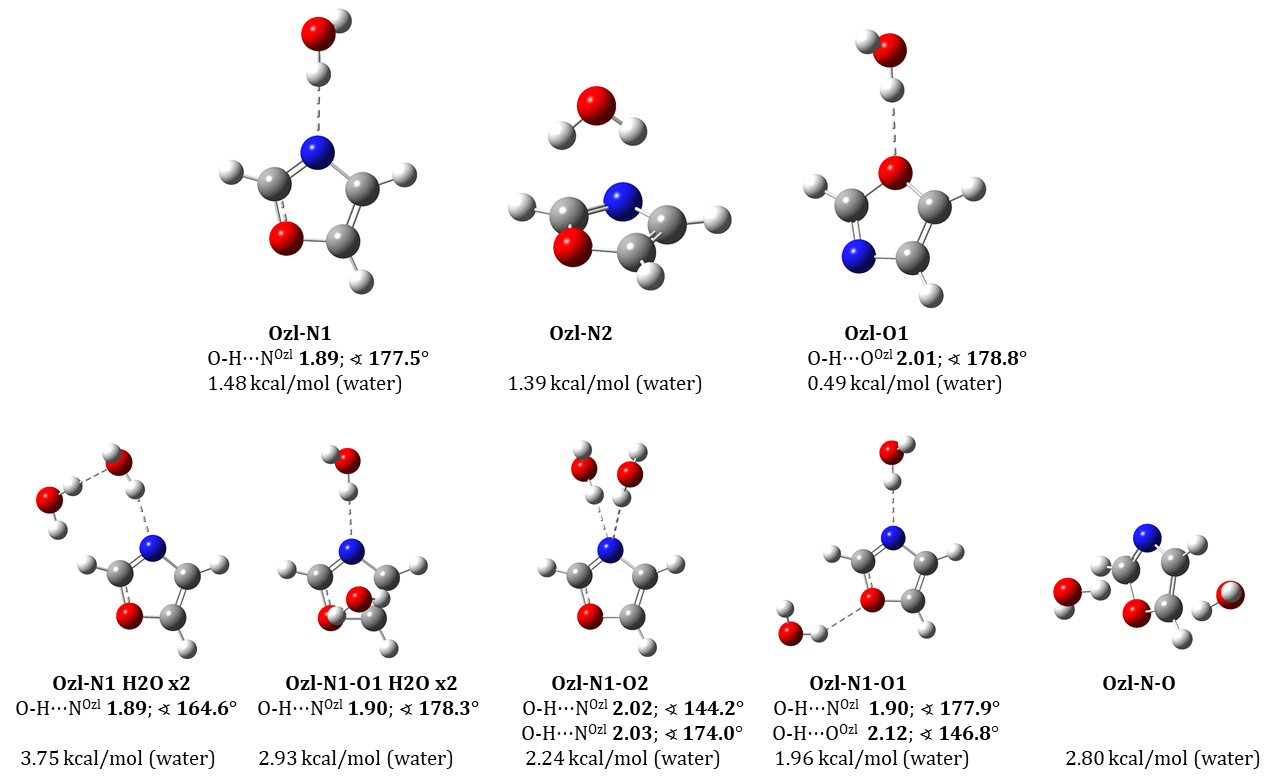


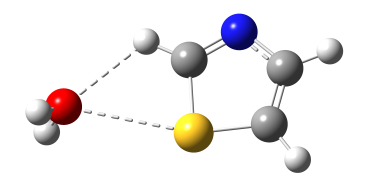


**Figure S2.** Optimized water-oxazole complexes after MD, selected parameters and interaction energies calculated in gas phase and water by M06-2X/6-311++G(d,p) method.

**Table S3**. Percentage of water molecules around the rings in the first solvation shell (2.5Å) retrieved form 200 snapshots form molecular dynamics for each ring.

| **Number of water molecules** | **Thiazole [%]** | **Oxazole [%]** |
| --- | --- | --- |
| 0 | 14.0 | 5.5 |
| 1 | 48.0 | 35.0 |
| 2 | 35.5 | 44.0 |
| 3 | 2.5 | 13.0 |
| 4 | 0.0 | 2.5 |


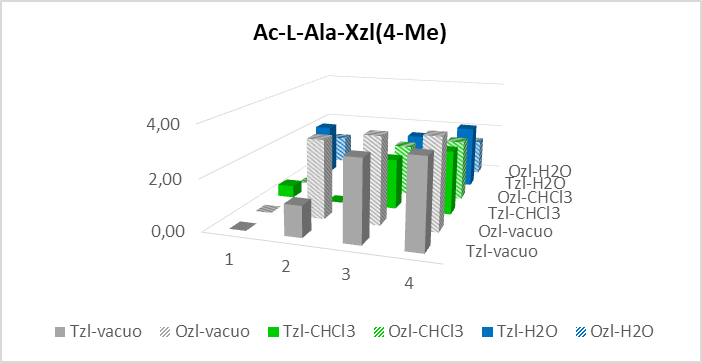


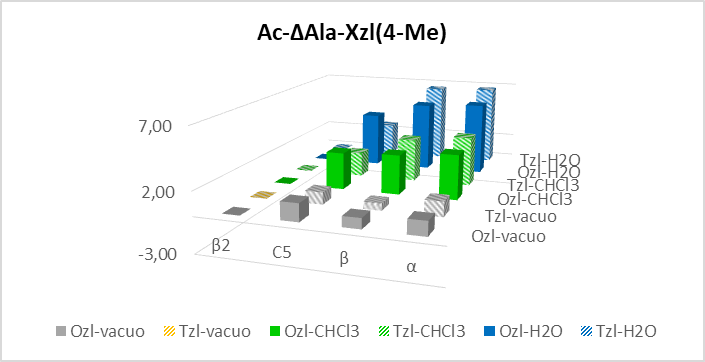

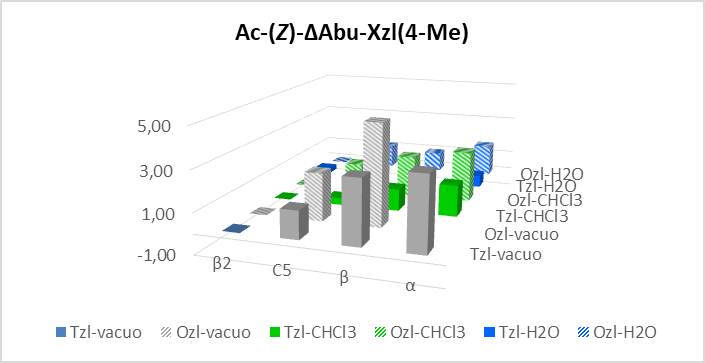


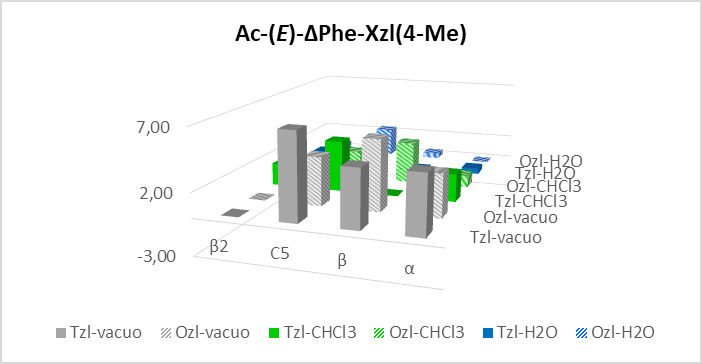

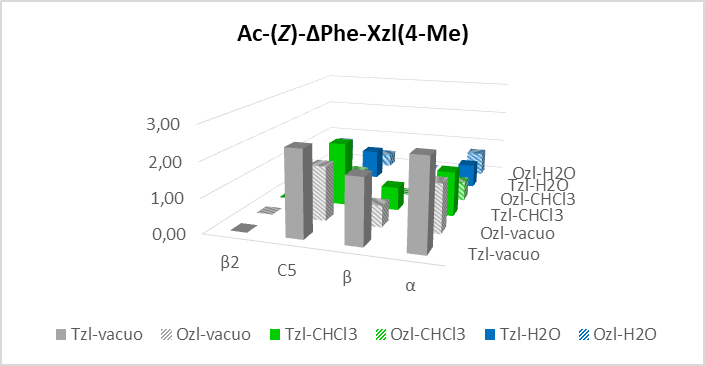


**Figure S3.** The comparison of relative energy between oxazole- and thiazole-amino acid residues with the same side chain.

**Reference**

1. Vargas, R., et al., Conformational study of the alanine dipeptide at the MP2 and DFT levels. Journal of Physical Chemistry A, 2002. **106**(13): p. 3213-3218.
